# Enhanced contrast acoustic‐resolution photoacoustic microscopy using double‐stage delay‐multiply‐and‐sum beamformer for vasculature imaging

**DOI:** 10.1002/jbio.201900133

**Published:** 2019-08-07

**Authors:** Moein Mozaffarzadeh, Mehdi H. H. Varnosfaderani, Arunima Sharma, Manojit Pramanik, Nico de Jong, Martin D. Verweij

**Affiliations:** ^1^ Department of Imaging Physics, Laboratory of Acoustical Wavefield Imaging Delft University of Technology Delft The Netherlands; ^2^ Department Biomedical Engineering Thoraxcenter, Erasmus MC Rotterdam The Netherlands; ^3^ Department of Biomedical Engineering Tarbiat Modares University Tehran Iran; ^4^ School of Chemical and Biomedical Engineering Nanyang Technological University Singapore Singapore

**Keywords:** acoustic‐resolution photoacoustic microscopy, contrast enhancement, synthetic aperture focusing technique, vasculature imaging, virtual source

## Abstract

In acoustic‐resolution photoacoustic microscopy (AR‐PAM) systems, the lateral resolution in the focal zone of the ultrasound (US) transducer is determined by the numerical aperture (NA) of the transducer. To have a high lateral resolution, a large NA is used. However, the larger the NA, the smaller the depth of focus [DOF]. As a result, the lateral resolution is deteriorated at depths out of the focal region. The synthetic aperture focusing technique (SAFT) along with a beamformer can be used to improve the resolution outside the focal region. In this work, for image formation in AR‐PAM, we propose the double‐stage delay‐multiply‐and‐sum (DS_DMAS) algorithm to be combined with SAFT. The proposed method is evaluated experimentally using hair targets and in vivo vasculature imaging. It is shown that DS_DMAS provides a higher resolution and contrast compared to other methods. For the B‐mode images obtained using the hair phantom, the proposed method reduces the average noise level for all the depths by about 134%, 57% and 23%, compared to the original low‐ resolution, SAFT+DAS and SAFT+DMAS methods, respectively. All the results indicate that the proposed method can be an appropriate algorithm for image formation in AR‐PAM systems.

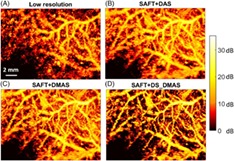

## Graphical abstract

For image formation in acoustic‐resolution photoacoustic microscopy, double‐stage delay‐multiply‐and‐sum (DS_DMAS) beamforming algorithm is introduced. The proposed method is experimentally evaluated for in vivo vasculature imaging. As shown in the figure, DS_DMAS provides a higher resolution and contrast compared to other methods. (D) Has a lower average noise level for all the depths by about 134%, 57% and 23%, compared to A‐C, respectively.

## 1 INTRODUCTION

Photoacoustic imaging (PAI) is a promising imaging modality which provides structural, molecular and functional information. In PAI, a light source irradiates the tissue. Then, the subsequent thermal gradient induces wide‐band acoustic waves based on the thermoelastic effects 1, 2. These waves are recorded by an ultrasound (US) transducer. The main advantage in PAI is to have both the optical contrast and US resolution in a single imaging modality. Compared to pure optical imaging, PAI provides a better resolution at more than one optical transport mean‐free‐path (more than ∼1 mm) due to the fact that the US waves get scattered 2‐3 orders of magnitude less than the optical waves 3, 4. On the other hand, US imaging is based on reflections due to local differences in the mechanical properties of tissues, which makes it hard to detect early tumors. PAI addresses this problem since it is based on optical absorption, and the final photoacoustic (PA) image is the optical absorption distribution map of tissue 5, 6.

Over the past decade, many papers have been published indicating different applications of PAI, for example, breast imaging 7, ocular imaging 8, 9, cancer detection and staging 6 and sentinel lymph node (SLN) imaging 2, 10, 11. Exogenous contrast agents have also been helpful during the development of PAI imaging systems 12, 13, 14. Based on the imaging configuration, PAI systems are categorized into two groups: photoacoustic tomography 15, 16 and photoacoustic microscopy (PAM) 17, 18. PAM is separated into two categories: acoustic‐resolution PAM (AR‐PAM) and optical‐resolution PAM (OR‐PAM). In AR‐PAM, the resolution is determined by the focus of the US transducer while in the OR‐PAM, the imaging resolution is determined by the optical focus 3, 19, 20. In this work, we focus on the AR‐PAM imaging systems. In addition, as the biomedical application, we focus on vasculature imaging. Clinically, noninvasive imaging of vasculature has a high value 3, 21, 22, 23, 24, 25, 26, 27. Tumors usually lead to a distorted vascular architecture around them. Studies in oncology also indicated that angiogenesis (formation/growth of new blood vessels) has a critical value in clarification of status of a tumor and possibility of metastasis. Inability to form new blood vessels might be related to the low metastatic activity 3, 28.

In PAM, usually a single element focused US transducer is used to detect the PA waves. Recording at one spatial coordinate of the XYZ system yields an A‐line (1D image). By sweeping the US transducer in one direction, B‐mode images (2D images) are obtained. In order to have 3D images, raster scanning is needed in both the X and Y directions (assuming the Z direction indicates depth). Once the imaging medium is fully covered, 3D images or maximum amplitude projection (MAP) can be used to visualize the optical absorption distribution of the imaging target 29.

**Figure 1:**
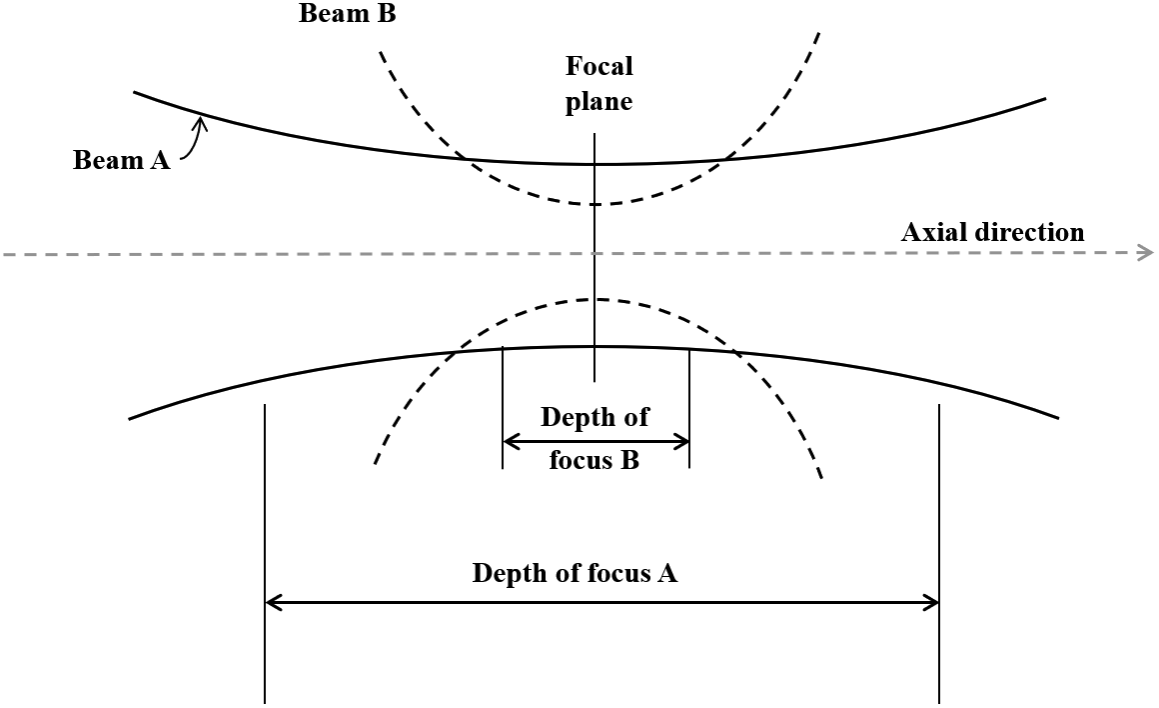
Comparison of depth‐of‐focus and beamwidth for a large and small numerical aperture

The general method for image formation in PAM is putting all the A‐lines next to each other to form a 2D/3D image. In AR‐PAM, imaging resolution depends on the properties of the focused US transducer. The lateral resolution is calculated by *R*_*L*, *AR* − *PAM*_ = 0.71*c*_0_/*NAf*_0_, where *c*_0_, *NA*, and *f*0 are the sound speed, numerical aperture (NA), and PA central frequency, respectively 1. To have a high resolution, a large NA is desired. However, a large NA decreases the focal region (DOF) 30, 31. This is explained in Figure 1 where the beam B has a narrower beamwidth at the focal plane (indicating a higher lateral resolution compared to beam A), but its DOF is shorter than beam A. In such a condition, even though the lateral resolution in the focal zone (or plane) is improved, it gets deteriorated outside the focal region of the US transducer. Consequently, out‐of‐focus (out of DOF), the image quality is significantly reduced. To address this problem, the synthetic aperture focusing technique (SAFT) using the virtual detector (VD) concept was proposed for both US and PA imaging systems 32, 33, 34. This method was also used for the in vivo imaging scenario in a PAM system 3. In this method, it is assumed that the A‐lines are detected at the VD location, which is the location of the focus of the US transducer. Then, new A‐lines are synthetically generated by combining properly delayed versions of the detected A‐lines. The 2D SAFT 35 and adaptive SAFT 36 are the expanded versions of the conventional SAFT 32, 33, 34.

For the synthesis of new A‐lines, a beamformer is used. Usually, the delay‐and‐sum (DAS) beamformer (due to its simple implementation) is utilized to obtain the new A‐lines. However, as demonstrated in previous publications, DAS is a blind beamformer which treats all the input A‐lines equally. Consequently, it leads to a high noise level (low contrast) and a wide beam (low resolution). Different adaptive beamformers and weighting factors have been developed to address the incapabilities in DAS 37, 38, 39, 40. One of the methods is delay‐multiply‐and‐sum (DMAS) which first was used for confocal microwave imaging (breast cancer detection) 41, and then for B‐mode US imaging 40. This algorithm was recently used in an AR‐PAM system to improve the image quality 42. We have previously reported an algorithm called double‐stage delay‐multiply‐and‐sum (DS_DMAS) 43, 44, 45, which outperforms DMAS in the terms of resolution and contrast.

In this work, due to the importance of vasculature, DS_DMAS algorithm has been used in an AR‐PAM system to have a higher image quality and vasculature distinguishability. Since DS_DMAS outperforms DAS and DMAS in macroscopic‐scale PAI scenarios, as demonstrated in 43, 44, we expect to obtain a higher image quality with DS_DMAS in microscopic‐scale PAI systems as well. DS_DMAS is utilized as the beamformer used in SAFT to synthesize the new A‐lines. We have experimentally evaluated the DS_DMAS algorithm; using hair‐target phantom and in vivo vasculature imaging. All the experimental results indicate that SAFT+DS_DMAS can be a proper algorithm to form images in AR‐PAM imaging systems.

The rest of the paper is as follows: in Section 2, SAFT, the proposed method and the experimental setup are explained. The results are presented in Section 3. Section 4 contains discussion regarding the methods and results. The conclusion is provided in Section 5.

## 2 MATERIALS AND METHODS

### 2.1 Conventional AR‐PAM

In conventional AR‐PAM, the detected A‐lines (1D data) are simply positioned next to each other, and at the end, a B‐mode image is generated by showing the formed 2D data. Once all the B‐mode images are arranged next to each other, then a 3D and/or MAP image can be generated. As mentioned, the problem with this method is that the lateral resolution is deteriorated 34.

### 2.2 SAFT and VD

**Figure 2:**
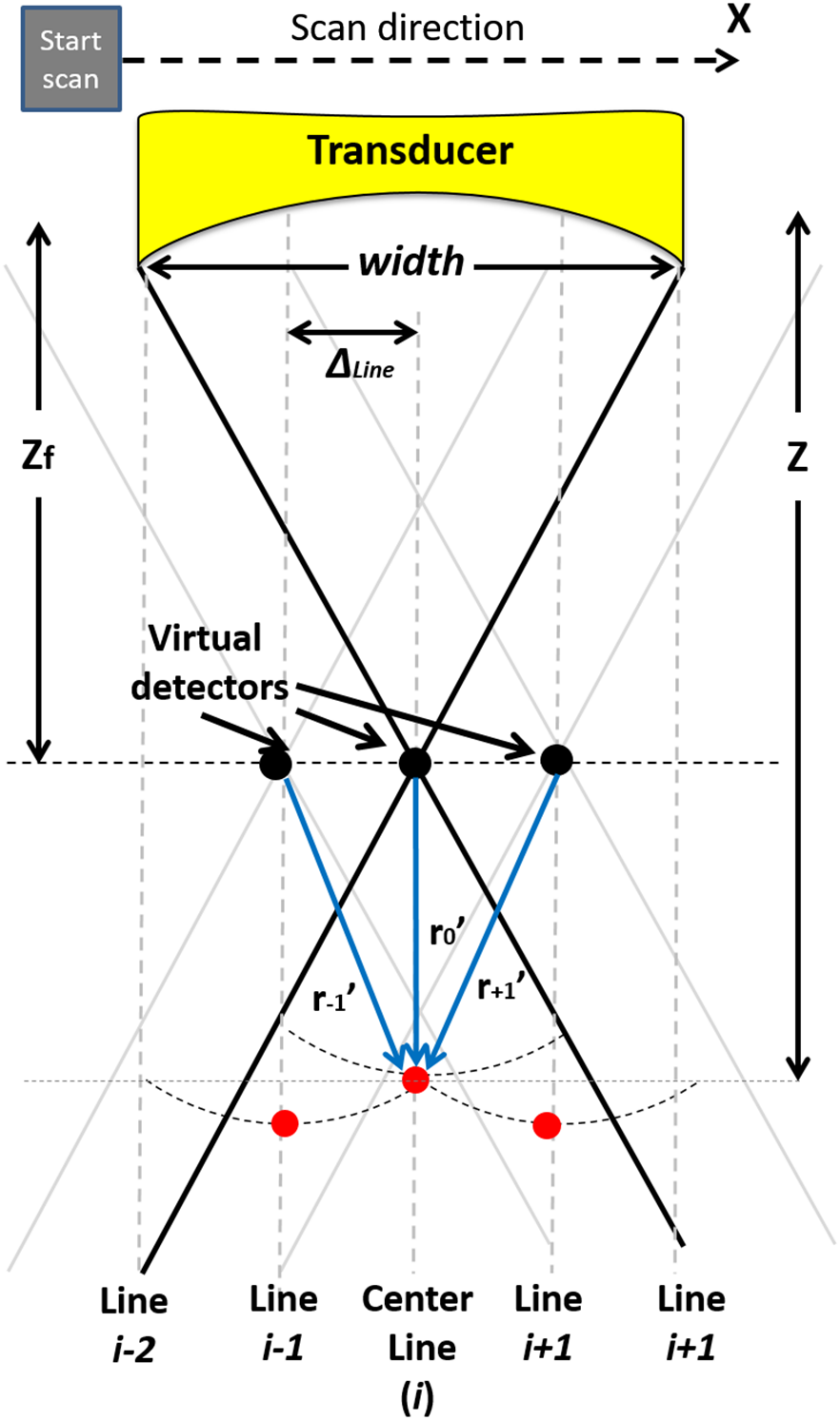
The schematic of the synthetic aperture focusing technique (SAFT) along with the geometry of the virtual detector (VD) to find the appropriate time delays used in SAFT. *z* is depth of the synthesized point, *z*_*f*_ is the depth of the VD, and *r*_*j*_ *'* is the distance between the VD of *j*th scan‐line next to the *i*th one and the synthesized point

The focal point of the US transducer is considered as the VD (see Figure 2). Due to the diverging of the beam away from the focus, adjacent PA signals will overlap during the image formation procedure. As a result, a higher quality image is formed, compared to conventional method 3, 33, 34. In other words, a set of new A‐lines is generated by combining the detected A‐lines and adjusting them based on the location of the VD and the synthesized imaging point.

The schematic presented in Figure 2 explains the way in which the intensity of a pixel in the final image can be obtained. As seen, the scan‐lines in the vicinity of the synthesized pixel are used. At each location of the transducer, we have a laser excitation. Then, a time series of the PA waves are detected by the US transducer. This is considered as a low‐resolution A‐line. The A‐lines are used as the input of the SAFT algorithm. In SAFT, it is assumed that the reflection from a synthesized point on the axis of the US transducer is also manifest of the detected A‐lines next to this point (see Figure 2, red point in lines *i* − 1 and *i* + 1 have some information about the red point at line *i*). Therefore, by applying proper delays, the signals from these A‐lines can be made to overlap in the synthesized point. For this purpose, beamformers can be used.

Based on Figure 2, to form a high‐resolution pixel at the depth *z* = *ct* and the *i* ^*th*^ scan‐line, the following equation can be used:

(1) $$y_{\text{SAFT}+\mathrm{DAS}}\left(i,t=z/c\right)=\frac{1}{2N_{D}+1}\sum_{j=-N_{D}}^{N_{D}}\mathrm{RF}\left(i+j,t-\Delta t_{j}\right),$$

where RF(*i*, *t*) is the received zero‐mean signal at the *i*th scan‐line. *N*_*D*_ is the number of adjacent scan‐lines (next to the *i*th scan‐line) that contribute to the pixel. This number is determined by the opening angle of the transducer. Δ*t*_*j*_ is the time delay applied to the received signal of the *j*th scan‐line next to the *i*th scan‐line. The Δ*t*s for each scan‐line are calculated based on the distance between the VD of scan‐line and the synthesized pixel:

(2) $$\Delta t_{j}=\mathrm{sign}\left(z-z_{f}\right).\frac{\left(\left|z-z_{f}\right|-{r_{j}}^{\prime }\right)}{c},$$

where *c* is the speed of sound, *z* is the depth of the synthesized point, *z*_*f*_ is the depth of the VD, and *r*_*j*_ *'* is the distance between the VD of *j*th scan‐line next to the *i*th one and the synthesized point. Note that *r*_*j*_ *'* depends on *z*. The number of the involved scan‐lines, considering the opening angle of the transducer, is indicated based on the depth of the synthesized imaging point:

(3) $$N_{D}=\frac{\left|z-z_{f}\right|}{z_{f}}.\frac{w}{\Delta_{\text{Line}}},$$

where *w* is the width of the transducer, and Δ_Line_ is the separation between the scan‐lines. Eq. (1) describes the standard DAS beamforming in which a geometrical time delay is applied to the detected A‐lines, after which these are summed up. To overcome the blindness of DAS, DMAS was introduced in 40 for US imaging. The formula of the DMAS combined with SAFT can be written as follows:

(4) $$\;y_{\text{SAFT}+\text{DMAS}}\left(i,t\right)=\frac{2}{{\left(2N_{D}+1\right)}^{2}-\left(2N_{D}+1\right)}\sum_{j=-N_{D}}^{N_{D}-1}\sum_{k=j+1}^{N_{D}}{\hat{\mathrm{RF}}}_{\mathrm{jk}}\left(i,t\right),$$

where

(5) $$\begin{array}{l} {\hat{\mathrm{RF}}}_{\mathrm{jk}}\left(i,t\right)=S.\sqrt{\left|\mathrm{RF}\left(i+j,t-\Delta t_{j}\right)\mathrm{RF}\left(i+k,t-\Delta t_{k}\right)\right|,}\\ \;S=\mathrm{sign}\left(\mathrm{RF}\left(i+j,t-\Delta t_{j}\right)\mathrm{RF}\left(i+k,t-\Delta t_{k}\right)\right),\\ \;\text{for}-N_{D}\leq j<k\leq N_{D}. \end{array}$$

The superiority of DMAS, in comparison with DAS, is due to the fact that DMAS uses a correlation process to reduce the noise level. This leads to a higher image quality 42. The application of DAS and DMAS for AR‐PAM systems has been reported before 33, 34, 42. As evaluated in References 43, 44 for PAI and 45 for US imaging, DS_DMAS even outperforms DMAS, mainly in terms of noise level (and hence contrast). In this paper, we propose to combine DS_DMAS algorithm with SAFT for AR‐PAM systems. The DS_DMAS beamformer is generated from the expansion of the DMAS Equation 43, 44, and its formula combined with SAFT is as follows:

(6) $$y_{\text{SAFT}+\mathrm{DS}\_\text{DMAS}}\left(i,t\right)=\frac{2}{{\left(2N_{D}\right)}^{2}-2N_{D}}\sum_{j=-N_{D}}^{N_{D}-2}\sum_{k=j+1}^{N_{D}-1}{\tilde{\mathrm{rf}}}_{\mathrm{jk}}\left(i,t\right),$$

where

(7) $$\begin{array}{l} {\tilde{\mathrm{rf}}}_{\mathrm{jk}}\left(i,t\right)=S.\sqrt{\left|{\mathrm{rf}}_{j}\left(i,t\right).{\mathrm{rf}}_{k}\left(i,t\right)\right|}\\ \;S=\mathrm{sign}\left({\mathrm{rf}}_{j}\left(i,t\right).{\mathrm{rf}}_{k}\left(i,t\right)\right),\\ \;\text{for}-N_{D}\leq j<k\leq N_{D}-1, \end{array}$$

and

(8) $${\mathrm{rf}}_{f}\left(i,t\right)=\frac{1}{N_{D}-f}\sum_{k=f+1}^{N_{D}}{\hat{\mathrm{RF}}}_{\mathrm{fk}}\left(i,t\right).$$

Readers are referred to the References [43–45] for further information regarding this image formation method. In the Section 3, it will be shown that DS_DMAS combined with SAFT provides a higher contrast (lower level of noise) and subsequently, a better image quality.

### 2.3 Experimental study

#### 2.3.1 Imaging setup

**Figure 3:**
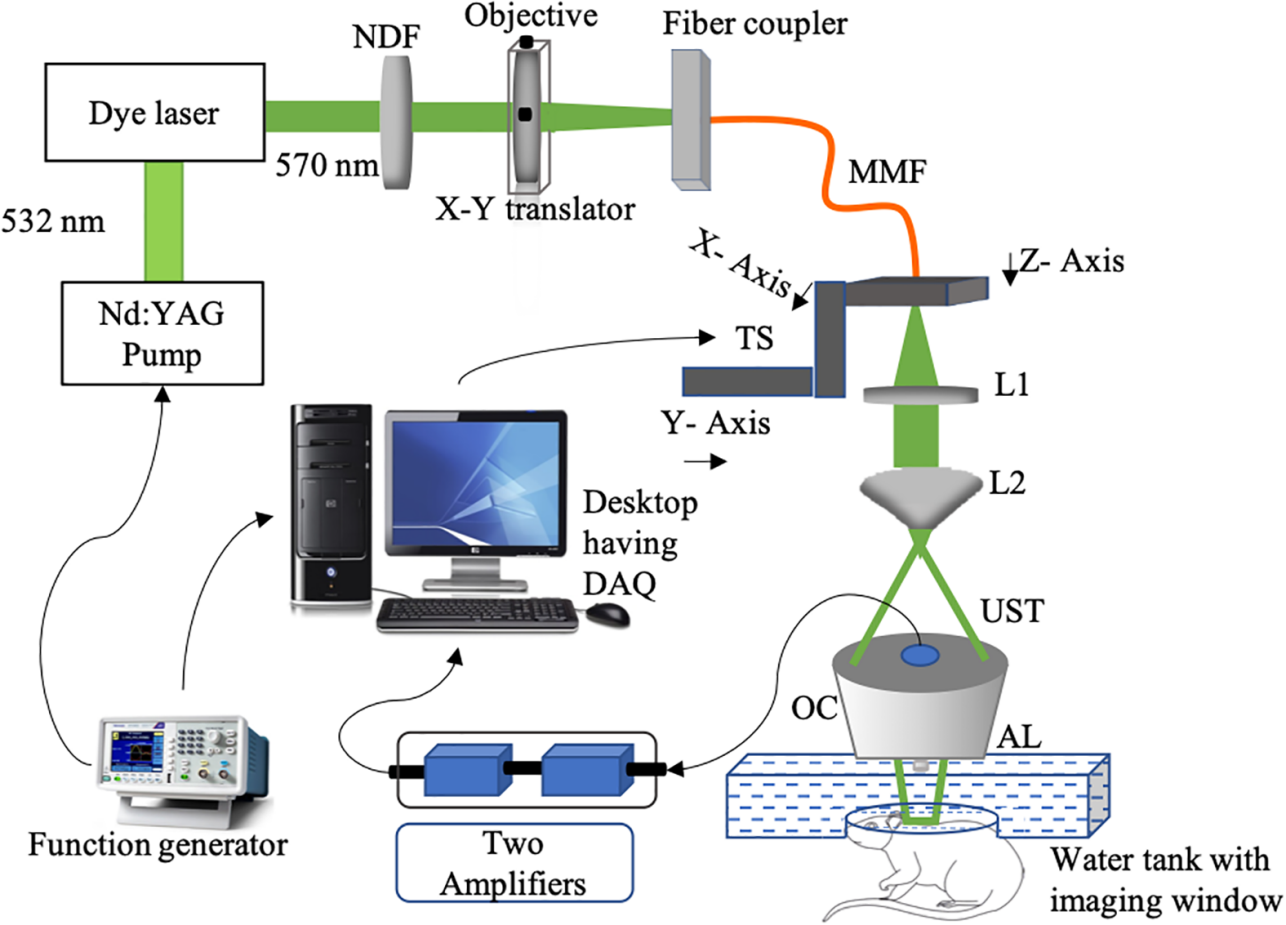
The schematic of the AR‐PAM system used for data acquisition. AL: Acoustic lens, AR‐PAM, acoustic‐resolution photoacoustic microscopy; DAQ, data acquisition card, L1, L2, lenses; MMF, multimode fiber; NDF, neutral density filter; OC, optical condenser; TS, translation stage; UST, ultrasound transducer

The imaging system used for data acquisition has been previously described in References 46, 47. The schematic of the AR‐PAM setup used for imaging is shown in Figure 3. Laser pulses with a wavelength of 570 nm and a pulse repetition rate (PRR) of 5000 Hz were generated using a nanosecond pulsed laser system consisting of a diode‐pumped solid‐state Nd‐YAG laser (INNOSLAB, Edgewave, Wurselen, Germany) and a dye laser (Credo‐DYE‐N, Sirah dye laser, Spectra Physics, Santa Clara, California). The laser beam was passed through a variable neutral density filter (NDF) (NDC‐50C‐4 M, Thorlabs), and focused on a multimode fiber (MMF) (M29 L01, Thorlabs) using an objective (M‐10X, Newport, Irvine, California) placed on an XY translator (CXY1, Thorlabs). The output beam was collimated using a collimating lens L1 (LA1951, Thorlabs), and then converted into a ring‐shaped beam by passing it through a conical lens L2 (1‐APX‐2‐B254, Altechna, Vilnius, Lithuania), having an apex angle of 130^°^. The resultant beam was directed to a homemade optical condenser (OC) having cone angles of 70^°^ and 110^°^. After total internal refection in the OC, the beam was refocused with an optical focal diameter of ∼2 mm. PA signal was generated on excitation of the sample by the resultant beam.

The reflected PA signal was collected using a 50 MHz ultrasound transducer (UST) (V214‐BB‐RM, Olympus‐NDT, Waltham, Massachusetts) housed in the center of the OC. An acoustic lens (AL) (LC 4573, Thorlabs) with a radius of curvature of 4.6 and a 6 mm diameter was attached to the UST using a UV curing optical adhesive (NOA61, Thorlabs). Thus, the acoustic focal spot was ∼46 μm. To maximize sensitivity, the optical excitation and acoustic detection were co‐focused such that the laser beam was loosely focused to the entire acoustic detection area. The scanning set‐up was mounted on a three‐axis motorized stage (TS)(PLS 85 for *x*‐ and *y*‐axis, VT 80 for *z*‐axis, PI‐Physik Instrumente, Karlsruhe, Germany), which was controlled by a three‐axis controller (SMC corvus eco, PI miCos GmbH, Germany). A water tank (13 × 30 cm) with a 10 cm imaging window sealed with polyethylene membrane, was placed directly below the scanning head. Two amplifiers each having 24 dB gain (ZFL‐500LN, Mini Circuits, Brooklyn, New York) amplified the PA signal before it was acquired by data acquisition (DAQ) card (M4i.4420, Spectrum, Grosshansdorf, Germany) installed in a desktop. The two channel DAQ card had a sampling rate of 250 Megasamples/second, 16 bit analog‐to‐digital converter, and 4 GB onboard memory. A function generator (GW INSTEK, AFG‐3051) triggered and synchronized the laser with DAQ. Data was acquired by performing two‐dimensional raster scanning of imaging head using Labview (National Instruments) as an interface.

#### 2.3.2 Hair sample preparation

Six horse hairs having an average diameter of 80 μm were arranged at different depths in 1% agar solution in a Petridish. After solidification of agar, the petridish was directly placed in the water tank below the scanning head. To get a B‐mode image containing all the 6 hair targets, multiple A‐lines were collected by moving the scanning head continuously in direction perpendicular to the hair samples. The speed of motor was maintained such the distance between two A‐lines was 3 μm.

#### 2.3.3 In vivo experiment

In vivo experiments were performed in accordance to the guidelines and regulations approved by the Institutional Animal Care and Use Committee of Nanyang Technological University, Singapore (Animal Protocol Number ARF‐SBS/NIE‐A0331). A 75 g adult female Sprague Dawley rat procured from InVivos Pte Ltd., Singapore, was used to image the vasculature surrounding the sentinel lymph node. The rat was anesthetized by intraperitoneal injection of 0.15 mL of cocktail containing ketamine and xylazine of dosage 85 and 15 mg/kg, respectively. During image acquisition, anesthesia was maintained by using vaporized isoflurane (1.2 L/min oxygen and 0.75% isoflurane, Medical Plus Pte Ltd., Singapore) delivered through custom made nose cone. The vitals of the animal were monitored using a pulse oximeter (Medtronic, PM1 0 N with veterinary sensor, Minneapolis, Minnesota) which was clipped to the hind‐paw.

Before imaging, the hair above the fore‐paw of the animal was depleted using commercially available hair removal cream. During imaging, the rat was placed on its side, such that the imaging area was directly below the polyethylene imaging window. Clear ultrasound gel was used as a coupling media to prevent any air gap between the rat and the imaging window.

A 12 × 15 mm^2^ area around the SLN of the rat was scanned to acquire images of the vasculature. Initially, the set up was arranged such that most of the vessels were in the focal plane of the transducer. Raster scanning was performed by moving the scan head in step sizes of 30 and 3 μm along *x*‐ and *y*‐axis, respectively. Next, the translation stage was moved twice with a step size of 1 mm along the Z‐axis to increase the distance between the UST and the imaging plane. The same area around the SLN was scanned after each step to get out‐of‐focus images of the vasculature. It took approximately 32 minutes for our experimental system to scan the entire area. After imaging, the rat was euthanized by injecting an overdose of pentobarbital.

## 3 RESULTS

### 3.1 B‐mode images

**Figure 4:**
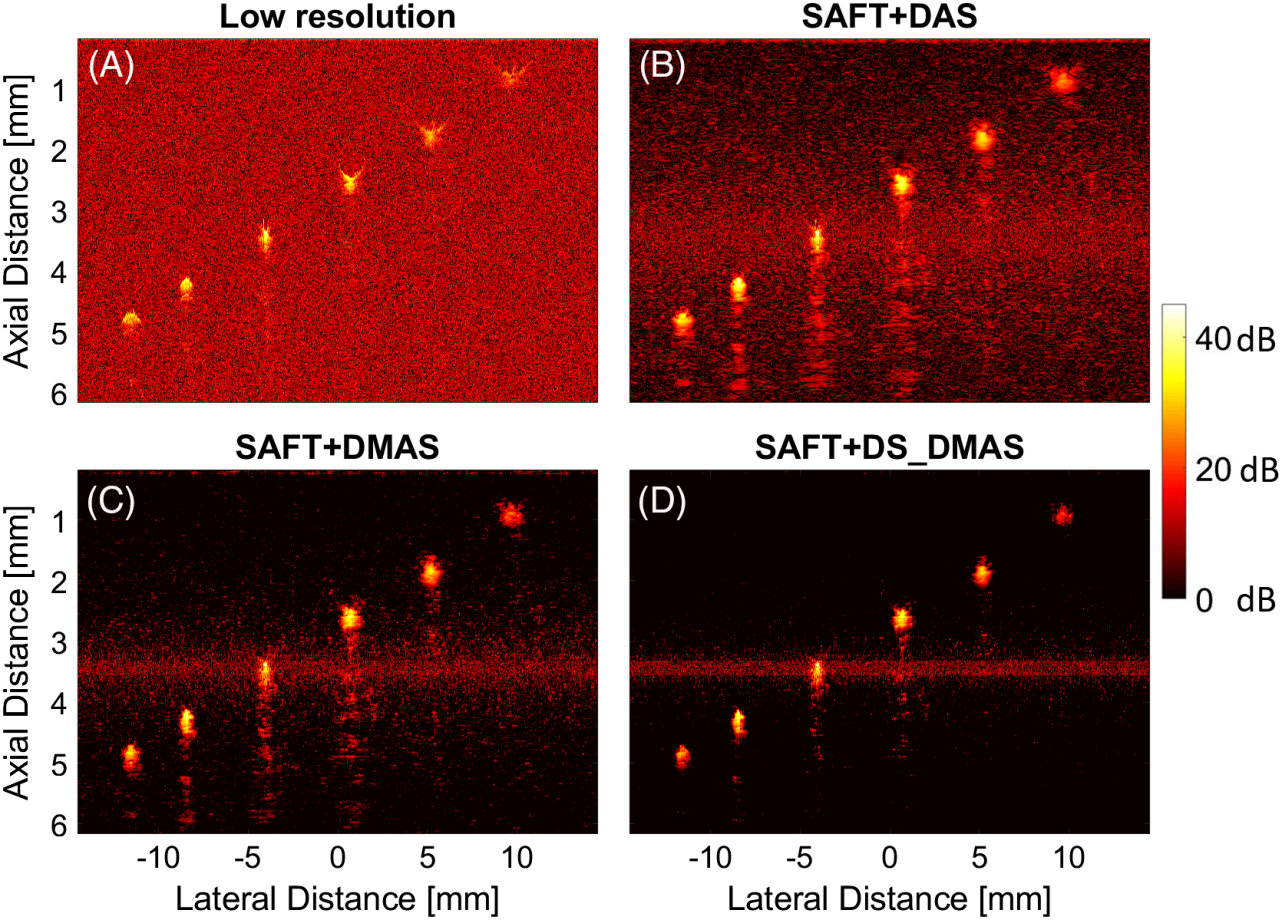
The B‐mode photoacoustic (PA) images reconstructed by (A) original low‐resolution A‐lines, (B) SAFT+DAS, (C) SAFT+DMAS and (D) SAFT+DS_DMAS methods. A B‐scan perpendicular to the hair targets is used as the input data. DAS, delay‐and‐sum; DMS, delay‐multiply‐and‐sum; DS, double‐stage; SAFT, synthetic aperture focusing technique

In this section, the performance of the proposed method and other methods is evaluated for B‐scans perpendicular to the hair targets. Therefore, it is expected to see cross‐sections of the targets; hairs will look like points in the reconstructed images in Figure 4.

The focal zone of the transducer is close to the third target from the left. Figure 4A shows that the original low‐resolution A‐lines used in AR‐PAM do not work well for targets outside the focal region of the US transducer. The targets, which are expected to be seen like a point, have an arc‐shape geometry far from the focal zone. Using the SAFT+DAS (Figure 4B), the detected signals are better aligned, and the arc‐shape geometry of the targets outside the focal region of the US transducer gets closer to a point. However, the background noise affects the image quality. SAFT+DMAS reduces the background noise (Figure 4C). As shown in Figure 4D, the degrading effects are even better suppressed by SAFT+DS_DMAS. The noisy line in the images obtained with SAFT is generated due to an inherent limitation of the VD method and SAFT, which is discussed in Section 4.

**Figure 5:**
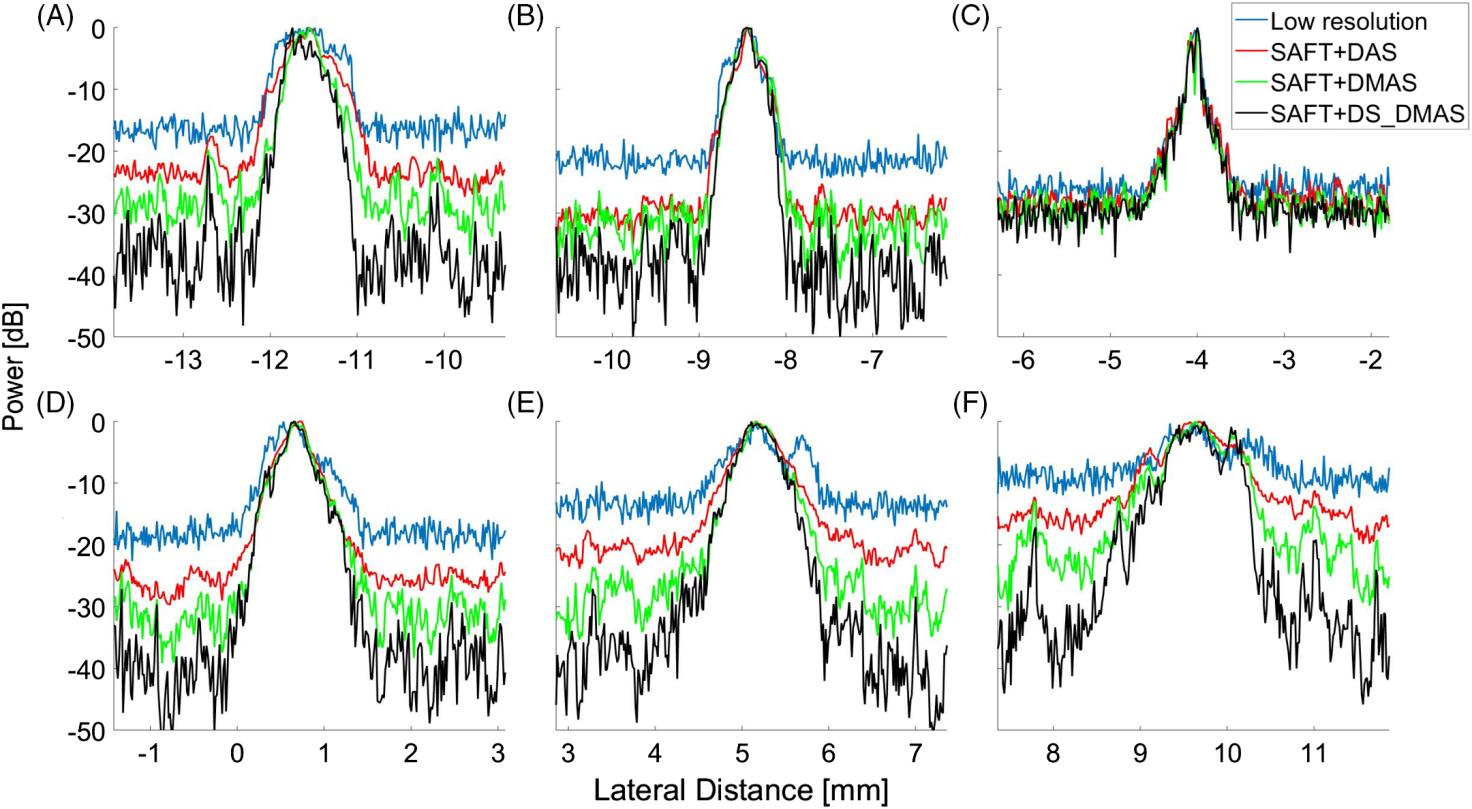
The lateral variations of the images shown in Figure 4. (A)‐(F) are related to the targets positioned at the depth of 4.85, 4.25, 3.42, 2.60, 1.83 and 0.91 mm, respectively; the point targets shown in Figure 4 from left to right

To better evaluate the improvements obtained by the proposed method, lateral variations of the six targets are presented in Figure 5. For all the targets, except the one in the focal zone of the transducer (Figure 5C), the proposed method outperforms the other methods mainly because the lower noise level results in a higher contrast in the images.

**Figure 6:**
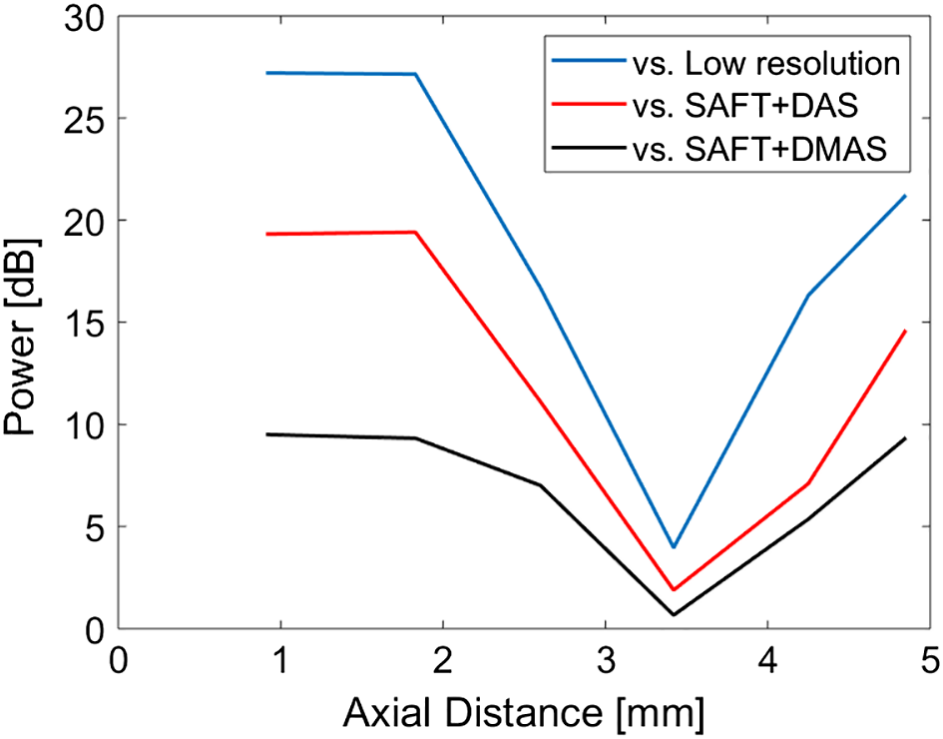
The noise level improvements obtained by SAFT+DS_DMAS compared to the other methods, at different depths. DMS, delay‐multiply‐and‐sum; DS, double‐stage; SAFT, synthetic aperture focusing technique

To quantitatively evaluate the methods, the noise level is presented in Table 1. The amplitudes of the noise level in the first 0.75 mm in Figure 5 are averaged linearly, and this average is presented in dB in Table 1. The noise level is calculated for all the targets which are positioned in different imaging depths. For all the depths, SAFT+DS_DMAS outperforms other methods. For instance, at the depth of 4.85 mm, which is out of the focal zone of the transducer, SAFT+DS_DMAS reduces noise level about 21.2, 14.6 and 9.3 dB, compared to the original, SAFT+DAS and SAFT+DMAS, respectively. Figure 6 shows the improvements obtained by SAFT+DS_DMAS at difference depths. The obtained improvement is low around the focal point of the transducer.

**Table 1:** Noise level (in dB) of the considered imaging methods at different depths for the B‐mode images shown in Figure 4

Noise level (dB)				
Depth (mm)	Low res	SAFT+DAS	SAFT+DMAS	SAFT+DS_DMAS
4.85	−16.5	−23.1	−28.4	−37.7
4.25	−21.8	−31.0	−32.7	−38.1
3.42	−26.5	−28.5	−29.8	−30.4
2.60	−18.6	−24.2	−28.3	−35.3
1.83	−13.5	−21.4	−31.5	−40.8
0.91	−09.0	−16.8	−26.7	−36.2

Abbreviations: DAS, delay‐and‐sum; DMS, delay‐multiply‐and‐sum; DS, double‐stage; SAFT, synthetic aperture focusing technique.

### 3.2 Maximum amplitude projection

**Figure 7:**
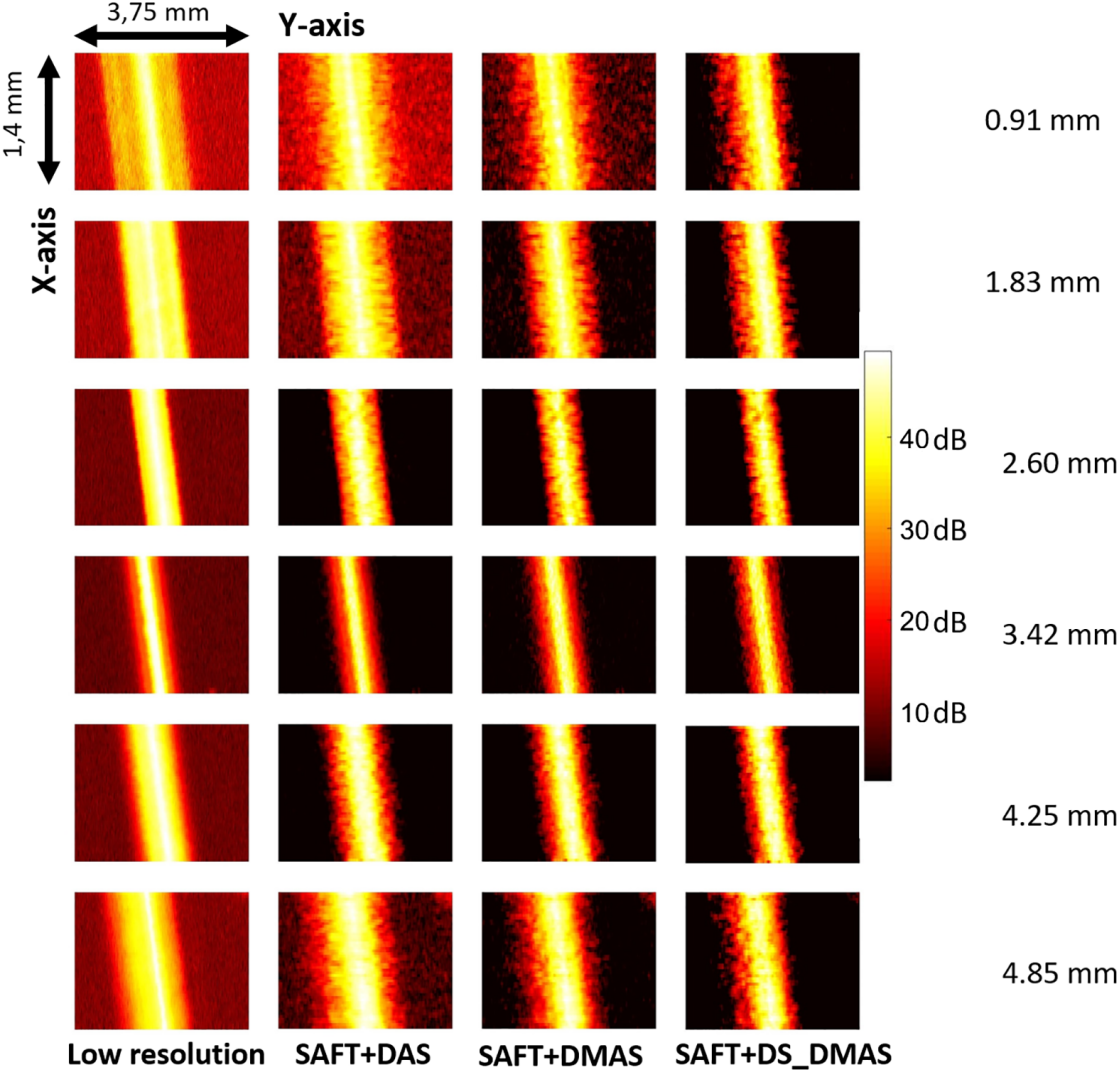
The maximum amplitude projection (MAP) of the hair‐target phantom. The numbers on the right side indicate the depth of the hairs

In order to evaluate the performance of the proposed method in 3D, MAP is used. For the hair‐target phantom, the MAPs are shown in Figure 7 where SAFT+DS_DMAS provides a better‐degraded noise. In addition, the width of the detected hair targets gets narrower by SAFT+DS_DMAS, compared to other methods. For better evaluation, the lateral variations of the MAPs are presented in Figure 9 along with a quantitative evaluation for the noise level in Table 2. The amplitudes of the noise level in the last 0.75 mm in Figure 9 are averaged linearly, and this average is presented in dB in Table 2.

**Table 2:** Noise level (in dB) of the methods for maximum amplitude projection (MAP) images shown in Figure 7

Noise level (dB)				
Depth (mm)	Low resolution	SAFT+DAS	SAFT+DMAS	SAFT+DS_DMAS
0.91	−22.7	−35.4	−39.1	−53.4
1.83	−30.8	−42.6	−47.8	−60.1
2.60	−41.5	−55.2	−58.6	−68.0
3.42	−43.8	−51.8	−52.2	−59.3
4.25	−42.9	−53.7	−58.7	−67.6
4.85	−37.1	−45.4	−49.6	−61.0

Abbreviations: DAS, delay‐and‐sum; DMS, delay‐multiply‐and‐sum; DS, double‐stage; SAFT, synthetic aperture focusing technique.

**Figure 8:**
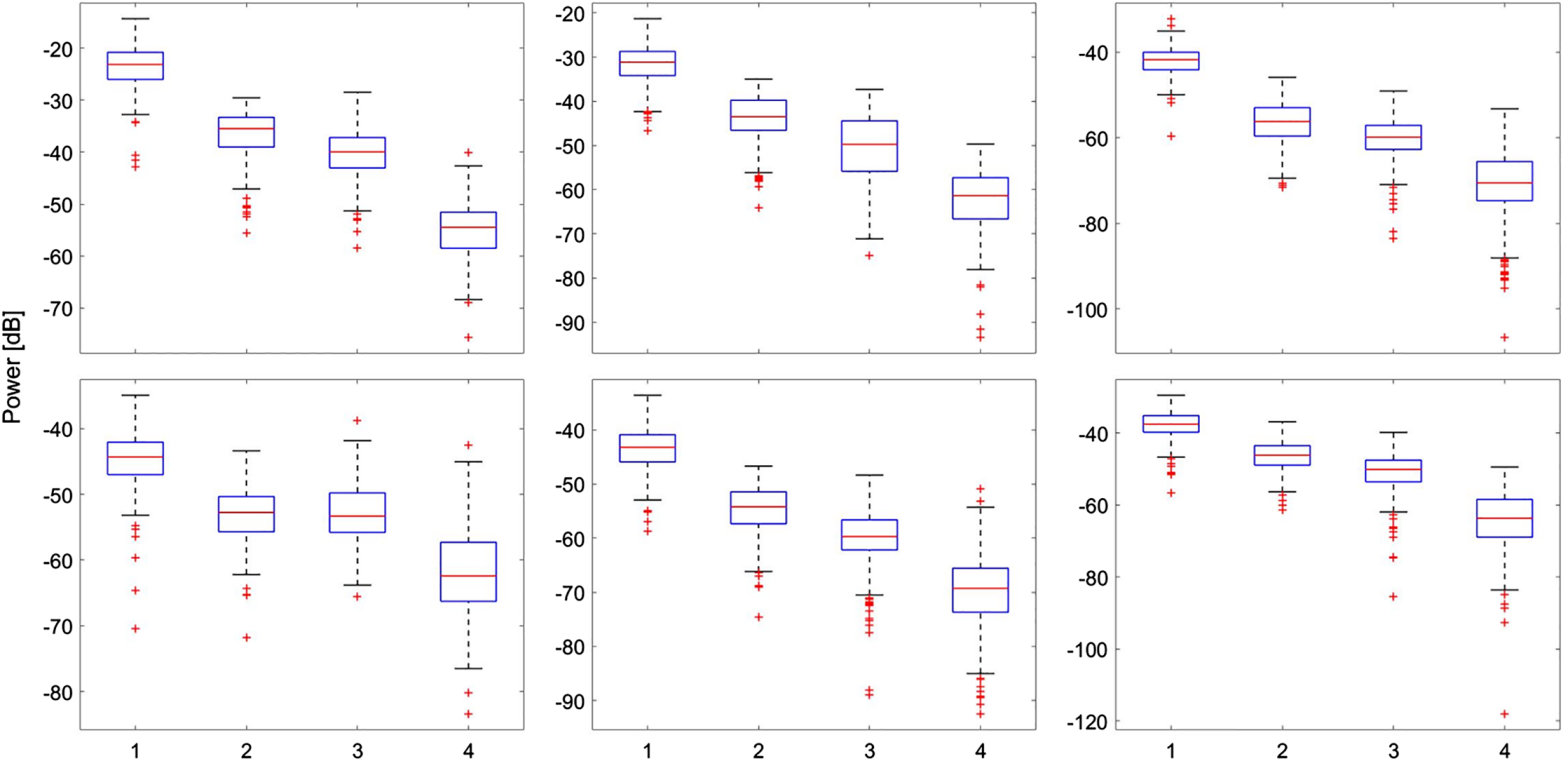
Statistical data analysis for the noise level of the graphs presented in Figure 9. The mean values are presented in Table 2. Columns 1–4 represents low‐resolution, SAFT+DAS, SAFT+DMAS and SAFT+DS_DMAS methods, respectively. A‐F, These are corresponding to Figure 9A‐F. DAS, delay‐and‐sum; DMS, delay‐multiply‐and‐sum; DS, double‐stage; SAFT, synthetic aperture focusing technique

**Figure 9:**
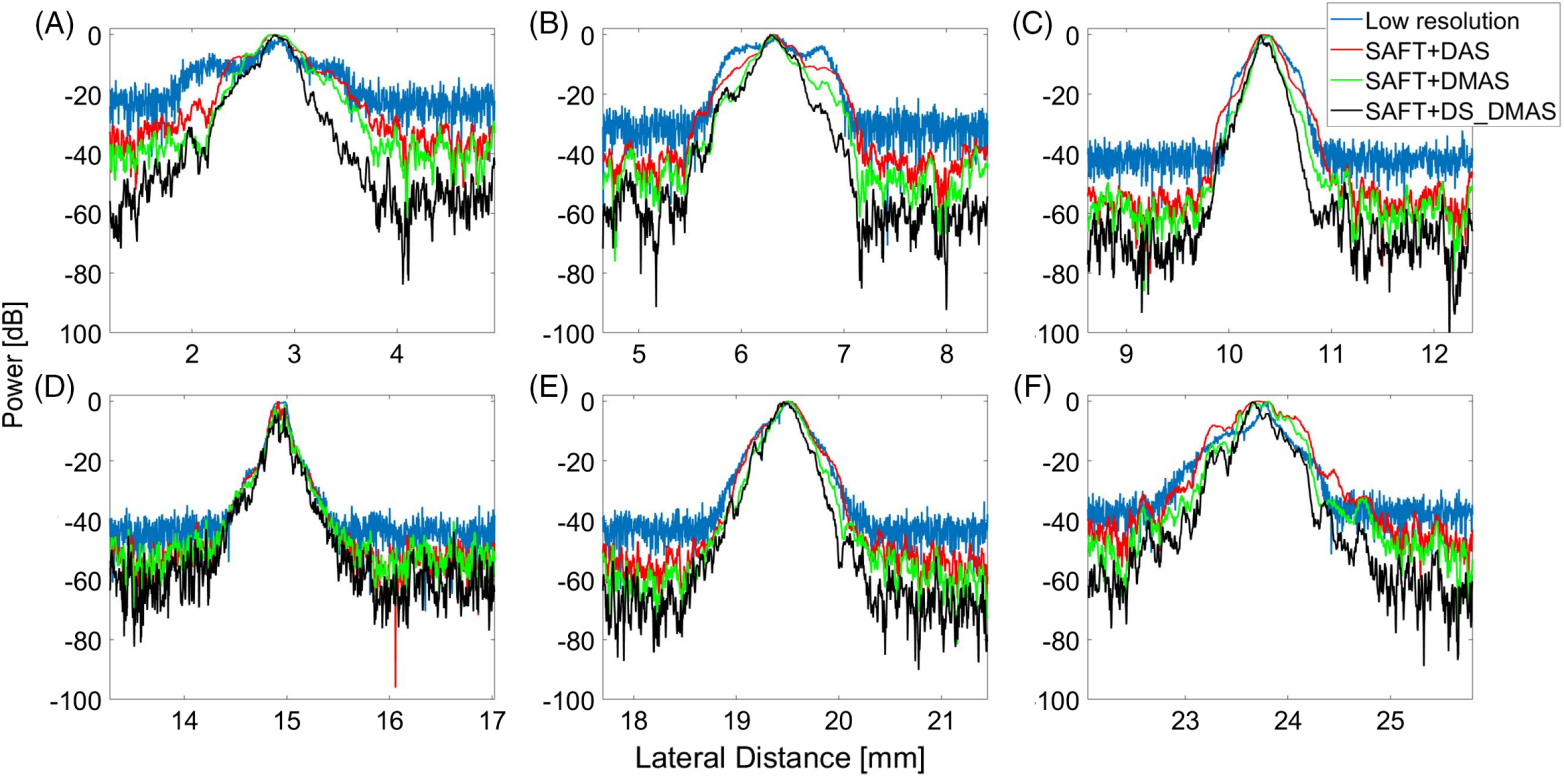
The lateral variations of the images shown in the Figure 7. (A)‐(F) These are related to the targets positioned at the depth of 0.91, 1.83, 2.60, 3.42 , 4.25 and 4.85 mm, respectively; each of which related to each row in the Figure 7

The lower noise level provided by the proposed method is reflected by the numbers presented in Table 2 where, for instance, at the depth of 0.91 mm (far from the focal point), the SAFT+DS_DMAS leads to lowering the noise level by 30.7, 18 and 14.3 dB, in comparison with the low‐resolution A‐lines, SAFT+DAS and SAFT+DMAS methods, respectively. In addition, considering the width of the mainlobe shown in Figure 9, it is clear that SAFT+DS_DMAS results in a narrower hair width, which indicates a better resolution. The adapted Figure 8 shows the statistical data analysis performed over the noise level of plots presented in Figure 9. On each box, the central mark indicates the median of the noise level inside the corresponding area. The bottom and top edges of the box indicate the 25th and 75th percentiles, respectively. This box shows the noise level of 50% of the lateral variations inside the corresponding area. The whiskers extend to the most extreme data points not considered outliers, and the outliers are plotted individually by the + symbol. It can be seen that the mean values (red line in the boxes) verify the values presented in Table 2. For all the depths, the SAFT+DAS and SAFT+DMAS provide distinguished boxes compared to the low‐resolution method. However, the boxes related to these two methods have overlapping. It can be seen that the box related to SAFT+DS_DMAS has a lower value and no overlapping, in comparison with other methods, for all the imaging depths.

### 3.3 In vivo imaging

**Figure 10:**
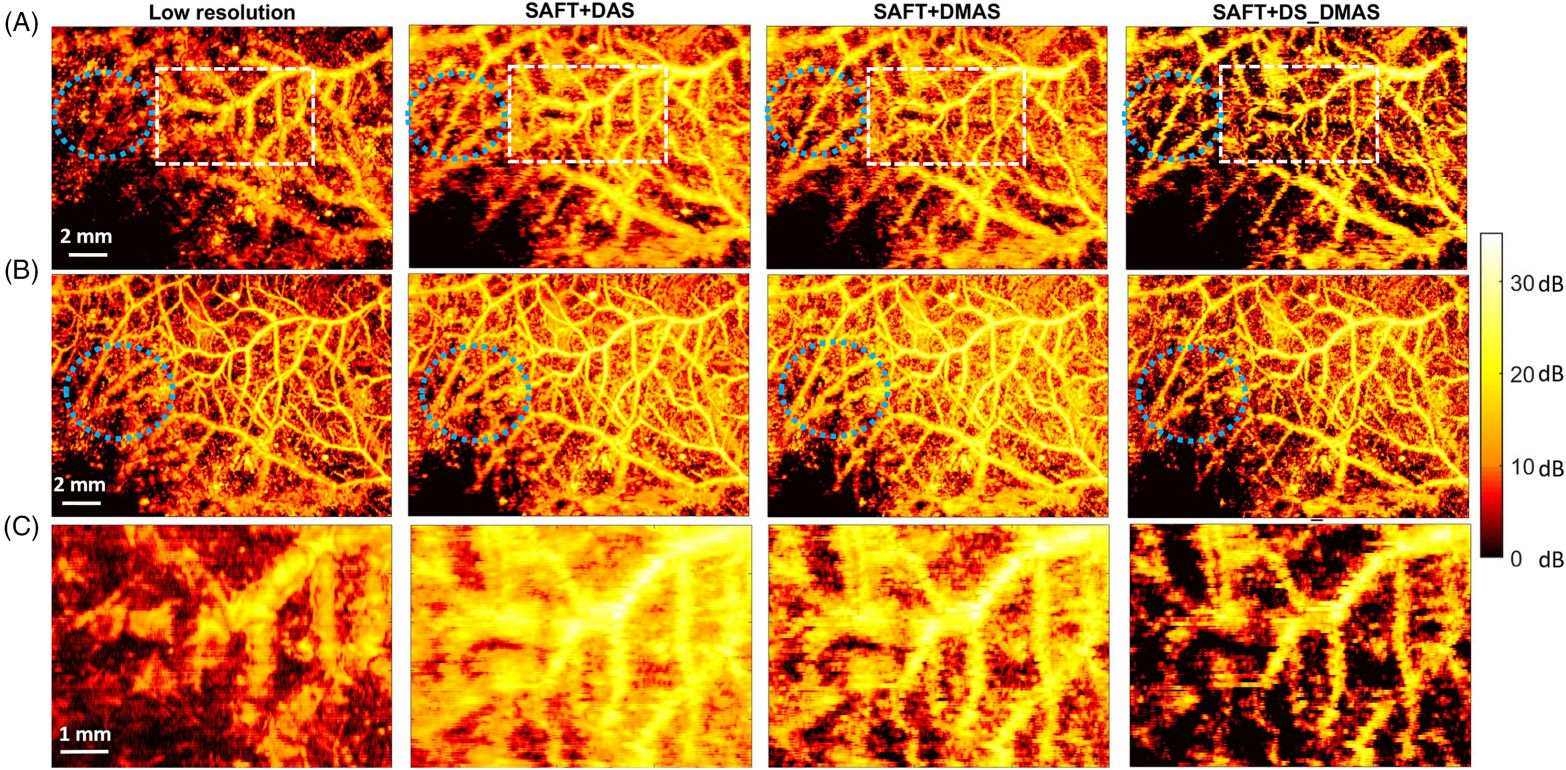
The maximum amplitude projection (MAP) images of the in vivo experiment. Most of vasculature are A, 1 mm below the focal point and B, at the focal point. C, A zoomed version of vasculature when Most of vasculature are 2 mm below the focal point of the transducer

The in vivo images with different methods of reconstruction are shown in Figure 10. In the first row, most of the absorbers (vasculature) are close to the focal point while in second row (Figure 10B), most of the absorbers are 1 mm below the focal point of the US transducer. Figure 10C represents a zoomed version of regions indicated by the white rectangles in Figure 10A while most of vasculature are 2 mm below the focal point of the transducer.

The higher image quality obtained by the proposed method can be seen by considering the vasculature inside the blue circles used in Figure 10A,B. In addition, comparing the area indicated by the white dashed rectangle in Figure 10A, it can be seen that the vasculature is better distinguished using the proposed method, and a darker background is obtained. This indicates a higher contrast.

## 4 DISCUSSION

Generally in PAM, the A‐lines are put next to each other to form a 2D image (B‐scan). In AR‐PAM, since a focused US transducer detects the PA waves, a high resolution is obtained in the focal plane of the transducer. In applications in which usually a very high resolution is needed, a large NA (or a high frequency) US transducer will be used (see Figure 1). This leads to a deteriorated lateral resolution out of the DOF of the transducer.

**Figure 11:**
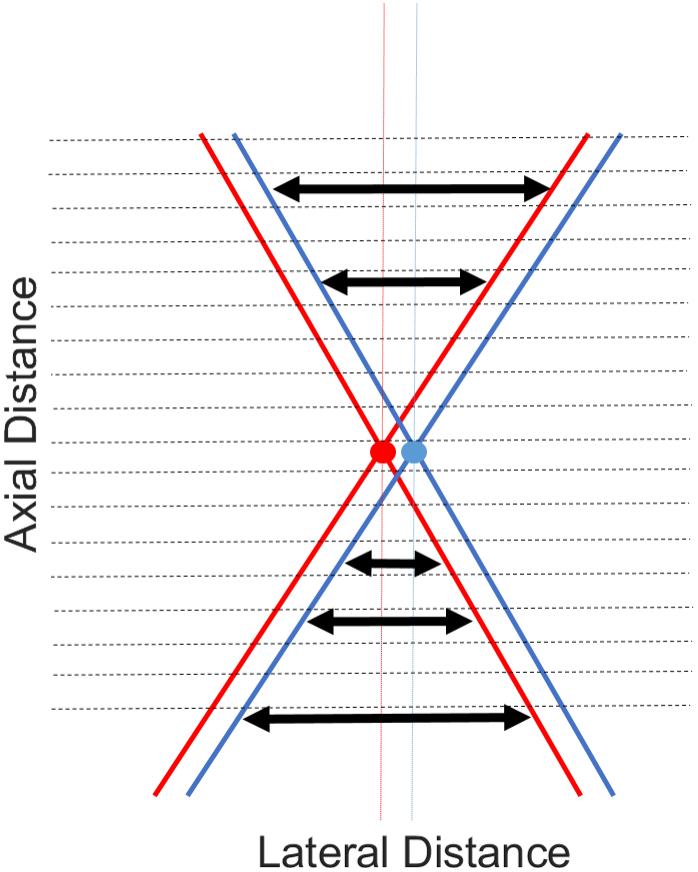
The effects of the distance between the depth of a synthesized imaging point and the virtual detector (VD)

To address this problem SAFT (using VD concept) was proposed in previous publications. Based on the angular extent of PA waves, SAFT assumes that each time sample in each A‐line (Line *i* in Figure 2.2) has projections in A‐lines in the vicinity of the line *i*, which is shown in Figure 2. In this case, based on the overlapping which exists between the triangular beam regions (above and below the VD), the synthetic high‐resolution A‐lines can be obtained. This method highly suppresses the background noise, as seen Figures 4 and 7. However, it should be noticed that this method does not remove imaging noise around the focal zone of the transducer (see the noisy area in Figure 4B). This so‐called noisy line is due to the fact that we have a limited overlapping between the triangular beam regions of VDs around the focal plane. To clarify this, consider Figure 11 where the different amount of overlapping vs depth is shown. Combining more observations in a single pixel involves some kind of averaging and will reduce the noise level. This can be perceived from Equation (3), where *N*_*D*_ is directly related to |*z* − *z*_*f*_|.

In AR‐PAM systems, beamformers should be used combined with SAFT and VD techniques. In this paper, it is proposed to use the SAFT method with the DS_DMAS beamformer to improve image quality. DS_DMAS is previously evaluated for macroscopic PAI systems 43, 44, 45, and its superiority over DAS and DMAS has been proved before. Here, for the first time, it is used for AR‐PAM. As proved by the lateral variations of the B‐scans and MAPs, shown in Figures 5 and 9, respectively, the proposed method provides lower noise level and better contrast. Lateral variations for the hair target located at the depth of 3.42 mm (Figure 5C) does not show a significant improvement since the focal plane of the US transducer is at the same depth. This depth, the original method already provides a high resolution and contrast. Therefore, using SAFT (ignoring the beamformer) does not significantly improve the image quality here. The proposed method does not improve the axial resolution in a noticeable manner while the range lobes are better suppressed (as seen in Figure 4) as a result of the higher noise suppression of DS‐DMAS algorithm.

The noise level is directly related to the image contrast. Tables 1 and 2 represent the noise level for the B‐mode and MAP images, respectively. As seen, the SAFT+DS_DMAS algorithm outperforms other methods. For the B‐mode images obtained using the hair phantom, the proposed method reduces the average noise level for all the depths by about 134%, 57% and 23%, compared to the original low‐resolution, SAFT+DAS and SAFT+DMAS methods, respectively. However, all the improvements achieved by the SAFT+DS_DMAS are obtained at the expense of a higher computational complexity. For each B‐mode image, with our software implementation, it takes 0.02, 1.40, 6.69 and 14.45 seconds by the original, SAFT+DAS, SAFT+DMAS and SAFT+DS_DMAS methods, respectively. The system for image formation contains an Intel(R) Core(TM) i7‐8650U *@* 1.90 GHz‐2.11 GHz processor and 8 GB of RAM. Recently, using implementation of the DS_DMAS on a Graphics Processing Unit (GPU) hardware, the processing time needed for DS_DMAS was reduced 48.

The results for the in vivo experiment (vasculature imaging) is presented in Figure 10. The higher performance of the proposed method can be appreciated by considering the background noise in the images and also the better distinguishability. The white rectangles in Figure 10A also shows the better separability of vasculature obtained by SAFT+DS_DMAS. It is also worth to mention that the proposed method provides a higher improvement when the targets are at a higher distance compared to the VD position, as indicated by Figure 6.

## 5 CONCLUSIONS

In AR‐PAM systems, to address the problem of the low resolution and contrast at regions outside the depth of focus of the US transducers, SAFT along with the VD was previously proposed by other researchers. In this paper, the DS_DMAS algorithm was used as the beamformer which works with SAFT to improve the contrast and resolution. The proposed method was evaluated experimentally using the hair‐target phantom and in vivo vasculature imaging. Both the B‐scans and MAP images were qualitatively and quantitatively evaluated. It was shown that the SAFT+DS_DMAS outperforms other methods in terms of contrast (affected by the noise level of an image) and resolution. For the images obtained from the hair phantom, SAFT+DS_DMAS reduces the average noise level for all the depths by about 18.95 dB, 12.28 dB and 7.56 dB, compared to the original low‐resolution, SAFT+DAS and SAFT+DMAS methods, respectively.

## CONFLICTS OF INTEREST

The authors declare no potential conflict of interests.

## AUTHOR BIOGRAPHIES

Please see Supporting Information online.

## Supporting information


**Author Biographies**

## References

[1] L. V. Wang , S. Hu , Science 2012, 335, 1458. 22442475 10.1126/science.1216210 PMC3322413

[2] M. Pramanik , K. Geng , C. Li , L. V. Wang , Med. Phys. 2008, 35, 2218. 18649451 10.1118/1.2911157 PMC2673632

[3] H. F. Zhang , K. Maslov , G. Stoica , L. V. Wang , Nat. Biotechnol. 2006, 24, 848. 16823374 10.1038/nbt1220

[4] V. Lihong , H.‐i. W. Wang , Biomedical Optics: Principles and Imaging, John Wiley & Sons, 2012.

[5] J. Yao , J. Xia , K. I. Maslov , M. Nasiriavanaki , V. Tsytsarev , A. V. Demchenko , L. V. Wang , Neuroimage 2013, 64, 257. 22940116 10.1016/j.neuroimage.2012.08.054 PMC3508393

[6] M. Mehrmohammadi , S. J. Yoon , D. Yeager , S. Y. Emelianov , Curr. Mol. Imag. 2013, 2, 89. 10.2174/2211555211302010010 PMC3769095 24032095

[7] L. Lin , P. Hu , J. Shi , C. M. Appleton , K. Maslov , L. Li , R. Zhang , L. V. Wang , Nat. Commun. 2018, 9, 2352. 29907740 10.1038/s41467-018-04576-z PMC6003984

[8] A. de La Zerda , Y. M. Paulus , R. Teed , S. Bodapati , Y. Dollberg , B. T. Khuri‐Yakub , M. S. Blumenkranz , D. M. Moshfeghi , S. S. Gambhir , Opt. Lett. 2010, 35, 270. 20125691 10.1364/OL.35.000270 PMC2886805

[9] A. Hariri , J. Lemaster , J. Wang , A. K. S. Jeevarathinam , D. L. Chao , J. V. Jokerst , Photoacoustics 2018, 9, 10. 29234601 10.1016/j.pacs.2017.11.001 PMC5723278

[10] T. N. Erpelding , C. Kim , M. Pramanik , L. Jankovic , K. Maslov , Z. Guo , J. A. Margenthaler , M. D. Pashley , L. V. Wang , Radiology 2010, 256, 102. 20574088 10.1148/radiol.10091772 PMC2897692

[11] R. Paridar , M. Mozaffarzadeh , V. Periyasamy , M. Pramanik , M. Mehrmohammadi , M. Orooji , Ultrasonics 2019, 96, 55. 31005780 10.1016/j.ultras.2019.03.010

[12] Z. Wang , P. K. Upputuri , X. Zhen , R. Zhang , Y. Jiang , X. Ai , Z. Zhang , M. Hu , Z. Meng , Y. Lu , et al., Nano. Res. 2019, 12, 49.

[13] M. F. Kircher , A. De La Zerda , J. V. Jokerst , C. L. Zavaleta , P. J. Kempen , E. Mittra , K. Pitter , R. Huang , C. Campos , F. Habte , et al., Nat. Med. 2012, 18, 829. 22504484 10.1038/nm.2721 PMC3422133

[14] P. Kanyi , A. J. Shuhendler , J. V. Jokerst , J. Mei , S. S. Gambhir , Z. Bao , J. Rao , Nat. Nanotechnol. 2014, 9, 233. 24463363 10.1038/nnano.2013.302 PMC3947658

[15] L. Yang , S. Park , F. Anis , S. Richarc , A. Oraevesky , M. Anastasio , IEEE Trans. Comput. Imag. 2019, 10.1109/TCI.2019.2895217.

[16] Q. Yuan , L. Li , Y. Shen , X. Wei , T. T. W. Wong , P. Hu , J. Yao , K. Maslov , L. V. Wang , Optica 2018, 5, 495. 30820444 10.1364/OPTICA.5.000495 PMC6388697

[17] T. T. W. Wong , R. Zhang , C. Zhang , H.‐C. Hsu , K. I. Maslov , L. Wang , J. Shi , R. Chen , K. K. Shung , Q. Zhou , et al., Nat. Commun. 2017, 8(1), 1386. 29123109 10.1038/s41467-017-01649-3 PMC5680318

[18] L. Lin , P. Zhang , S. Xu , J. Shi , L. Li , J. Yao , L. Wang , J. Zou , L. V. Wang , J. Biomed. Opt. 2016, 22, 041002. 10.1117/1.JBO.22.4.041002 PMC5075719 27775746

[19] S. Park , C. Lee , J. Kim , C. Kim , Biomed. Eng. Lett. 2014, 4, 213.

[20] L. V. Wang , IEEE J. Select. Topics Quant. Electron. 2008, 14(1), 171.

[21] X. Wang , K. Geng , M. A. Wegiel , D. J. Bornhop , G. Stoica , L. V. Wang , Opt. Lett. 2004, 29, 730. 15072373 10.1364/ol.29.000730

[22] K. Geng , X. Wang , X. Xie , G. Stoica , L. V. Wang , Appl. Opt. 2005, 44, 770. 15751858 10.1364/ao.44.000770

[23] X. Wang , Y. Pang , K. Geng , X. Xie , G. Stoica , L. V. Wang , Nat. Biotechnol. 2003, 21, 803. 12808463 10.1038/nbt839

[24] J. Yao , L. V. Wang , Laser Photon. Rev. 2013, 7, 758. 10.1002/lpor.201200060 PMC3887369 24416085

[25] Q. Zhang , Z. Liu , P. R. Carney , Z. Yuan , H. Chen , S. N. Roper , H. Jiang , Phys. Med. Biol. 2008, 53, 1921. 18364547 10.1088/0031-9155/53/7/008

[26] B. Rao , L. Li , K. Maslov , L. Wang , Opt. Lett. 2010, 35, 1521. 20479795 10.1364/OL.35.001521 PMC2966896

[27] J. G. Laufer , E. Z. Zhang , B. E. Treeby , B. T. Cox , P. C. Beard , P. Johnson , B. Pedley , J. Biomed. Opt. 2012, 17, 056016. 22612139 10.1117/1.JBO.17.5.056016

[28] P. Carmeliet , R. K. Jain , Nature 2000, 407, 249. 11001068 10.1038/35025220

[29] V. Periyasamy , N. Das , A. Sharma , M. Pramanik , J. biophotonics 2018, 12, e201800357. 10.1002/jbio.201800357 30511496

[30] T. L. Szabo , Diagnostic Ultrasound Imaging: Inside out, Academic Press, 2004.

[31] R. S. C. Cobbold , Foundations of Biomedical Ultrasound, Oxford University Press, 2006.

[32] C. H. Frazier , W. D. O'Brien , IEEE Trans. Ultrason. Ferroelectrics. Freq. Contr. 1998, 45, 196. 10.1109/58.646925 18244172

[33] M.‐L. Li , H. F. Zhang , K. Maslov , G. Stoica , L. V. Wang , Opt. Lett. 2006, 31, 474. 16496891 10.1364/ol.31.000474

[34] C.‐K. Liao , M.‐L. Li , P.‐C. Li , Opt. Lett. 2004, 29, 2506. 15584276 10.1364/ol.29.002506

[35] Z. Deng , X. Yang , H. Gong , Q. Luo , J. Appl. Phys. 2011, 109, 104701.

[36] Z. Deng , X. Yang , H. Gong , Q. Luo , Opt. Express 2012, 20, 7555. 22453434 10.1364/OE.20.007555

[37] M. Mozaffarzadeh , A. Mahloojifar , M. Orooji , K. Kratkiewicz , S. Adabi , M. Nasiriavanaki , J. Biomed. Opt. 2018, 23, 026002. 10.1117/1.JBO.23.2.026002 29405047

[38] M. Mozaffarzadeh , Y. Yan , M. Mehrmohammadi , B. Makkiabadi , J. Biomed. Opt. 2018, 23, 026005. 10.1117/1.JBO.23.2.026005 29446261

[39] M. Mozaffarzadeh , A. Mahloojifar , V. Periyasamy , M. Pramanik , M. Orooji , IEEE J. Sel. Top. Quant. Electron. 2019, 25(1), 1.

[40] G. Matrone , A. S. Savoia , G. Caliano , G. Magenes , IEEE Trans. Med. Imag. 2015, 34, 940. 10.1109/TMI.2014.2371235 25420256

[41] H. B. Lim , N. T. T. Nhung , E.‐P. Li , N. D. Thang , IEEE Trans. Biomed. Eng. 2008, 55(6), 1697. 18714833 10.1109/tbme.2008.919716

[42] J. Park , S. Jeon , J. Meng , S. Liang , J. S. Lee , C. Kim , J. Biomed. Opt. 2016, 21, 036010. 10.1117/1.JBO.21.3.036010 27020602

[43] M. Mozaffarzadeh , A. Mahloojifar , M. Orooji , S. Adabi , M. Nasiriavanaki , IEEE Trans. Biomed. Eng. 2018, 65, 31. 28391187 10.1109/TBME.2017.2690959

[44] M. Mozaffarzadeh , A. Hariri , C. Moore , J. V. Jokerst , Photoacoustics 2018, 12, 22. 30294542 10.1016/j.pacs.2018.09.001 PMC6171539

[45] M. Mozaffarzadeh , M. Sadeghi , A. Mahloojifar , M. Orooji , Ultrasound Med. Biol. 2018, 44, 677. 29276138 10.1016/j.ultrasmedbio.2017.10.020

[46] M. Moothanchery , M. Pramanik , Sensors 2017, 17, 357. 10.3390/s17020357 PMC5336060 28208676

[47] M. Moothanchery , A. Sharma , M. Pramanik , *J. Vis. Exp* 2017, 124, e55810. 10.3791/55810 PMC5608526 28671655

[48] S. R. M. Rostami, M. Mozaffarzadeh, M. Ghaffari‐Miab, A. Hariri, J. Jokerst. *Ultrason. Imag* 2019, 10.1177/0161734619862488. 31322057

