# Supplementary material for: Enhanced contrast acoustic‐resolution photoacoustic microscopy using double‐stage delay‐multiply‐and‐sum beamformer for vasculature imaging
Source: J Biophotonics. 2019 Aug 7;12(11):e201900133. doi: 10.1002/jbio.201900133 (PMC7065614; doi:10.1002/jbio.201900133)
Supplement: Supplementary file 1 — Author Biographies [file JBIO-12-e201900133-s001.docx]

| 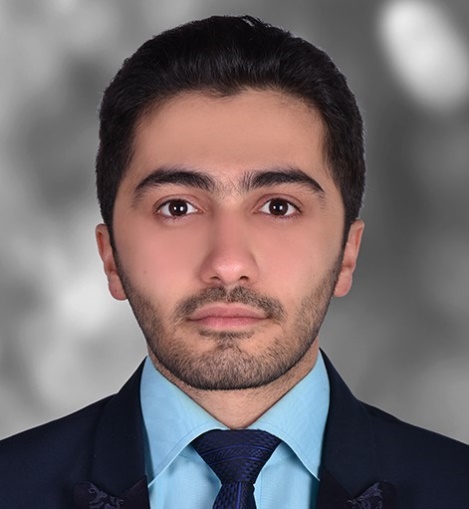 | **Moein Mozaffarzadeh** received his BSc degree in electrical engineering from Babol Noshirvani University of Technology, Mazandaran, Iran, in 2015, and his MSc degree in biomedical-bioelectric engineering from Tarbiat Modares University, Tehran, Iran, in 2017. He is currently a PhD student in the Department of Imaging Physics, Delft University of Technology. His research interests include photoacoustic/ultrasound image formation, photoacoustic endoscopy and intravascular photoacoustic imaging. |
| --- | --- |
| 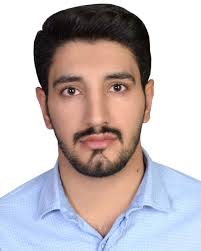 | **Mehdi Haji Heidari Varnosfaderani** received the B.Sc. degree in biomedical engineering from the Amirkabir University of Technology, Tehran, Iran, in 2013, and the M.Sc. degree in biomedical engineering from Tarbiat Modares University, Tehran, in 2016.  His research interests include biomedical signal processing, adaptive beamforming for medical ultrasound imaging, and ultrasound tissue harmonic imaging. |
| 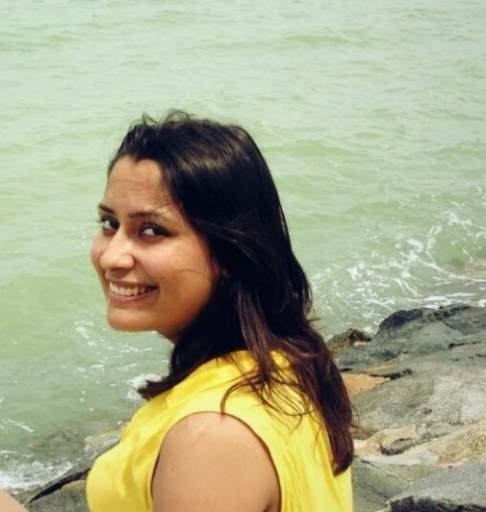 | **Arunima Sharma** received the Bachelor’s (integrated dual degree) degree in bioengineering and the master’s degree in biomedical technology both from the Indian Institute of Technology, Banaras Hindu University, Varanasi, India, in 2016. She is currently working toward the Ph.D. degree at the School of Chemical and Biomedical Engineering, Nanyang Technological University, Singapore.  Her current research interests include the development of photoacoustic imaging systems, and the application of photoacoustics in biomedical imaging. |
| 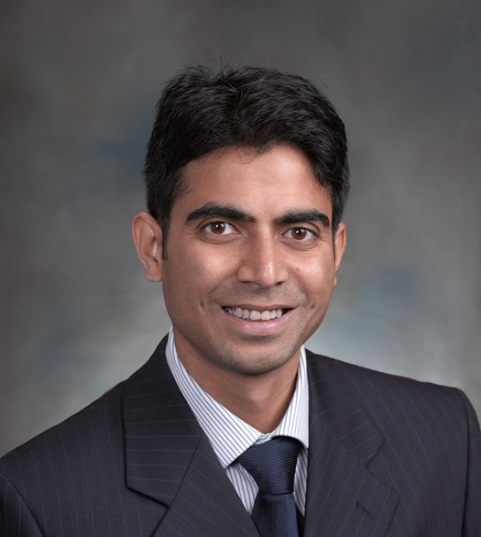 | **Manojit Pramanik** is currently an assistant professor at the School of Chemical and Biomedical Engineering, Nanyang Technological University, Singapore. He has received his PhD in biomedical engineering from Washington University in St. Louis, Missouri, USA. His research interests include the development of photoacoustic/thermoacoustic imaging systems, image reconstruction methods, clinical application areas, such as breast cancer imaging, molecular imaging, contrast agent development, and Monte Carlo simulation of light propagation in biological tissue. |
| 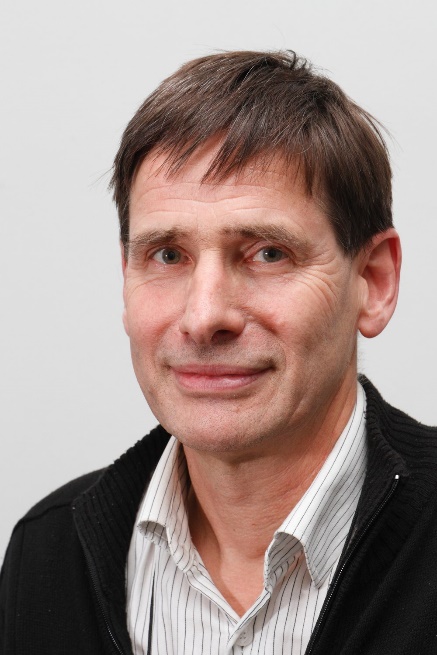 | **Nico de Jong** (A’97–M’09) graduated from the Delft University of Technology, Delft, The Netherlands, in 1978. He received the M.Sc. degree in physics (specialized in the field of pattern recognition) from the Delft University of Technology, and the Ph.D. degree from the Erasmus Medical Center, Rotterdam, The Netherlands, in 1993. His Ph.D. dissertation was on acoustic properties of ultrasound contrast agents.  Since 1980, he has been a Staff Member at the Thorax Center, Erasmus Medical Center. In 2003, he was a part-time Professor at the Physics of Fluids Group, University of Twente, Enschede, The Netherlands. He teaches on technical universities and the Erasmus Medical Center. He has been a Promotor of 29 Ph.D. students and is currently supervising 11 Ph.D. students. Since 2011, he has been a Professor in Molecular Ultrasonic Imaging and Therapy at the Erasmus Medical Center and Delft University of Technology.  Dr. de Jong is an Organizer of the Annual European Symposium on Ultrasound Contrast Imaging, held in Rotterdam and attended by approximately 175 scientists from universities and industries all over the world. He is on the safety committee of the World Federation of Ultrasound in Medicine and Biology. Over the last five years, he has given more than 30 invited lectures and has given numerous scientific presentations for international industries. He has been a guest editor for special issues of different journals. He is an Associate Editor of Ultrasound in Medicine and Biology. |
| 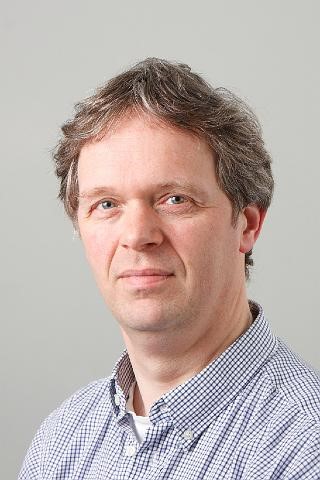 | **Martin D. Verweij** (M’10) received the M.Sc. (cum laude) and Ph.D. degrees in electrical engineering from Delft University of Technology, Delft, The Netherlands, in 1988 and 1992, respectively.  From 1993 to 1997, he was a Research Fellow with the Prestigious Royal Netherlands Academy of Arts and Sciences, Amsterdam, The Netherlands. In 1995 and 1997, he was a Visiting Scientist at Schlumberger Cambridge Research, Cambridge, U.K. In 1998, he became an Assistant Professor, and later an Associate Professor, with the Laboratory of Electromagnetic Research, Delft University of Technology, where he switched to the Laboratory of Acoustical Wavefield Imaging in 2011. Since 2015, he also has a part-time position at the Biomedical Engineering Group, Erasmus Medical Centre, Rotterdam, The Netherlands. His research interests include dedicated transducer design, beamforming algorithms, and the theoretical modeling and numerical simulation of medical ultrasound. He is the originator of the iterative nonlinear contrast source method for the computation of nonlinear ultrasound fields.  Dr. Verweij is a Research Leader of the Dutch Technology Foundation (STW) on projects involving transducer design, beamforming, and imaging. He was elected as the Best Teacher of the Electrical Engineering Curriculum in the academic year from 2001 to 2002. He is an Associate Editor of the Journal of the Acoustical Society of America, and a Treasurer of the Dutch Society for Medical Ultrasound. |
